# Overexpression of *ankyrin1* promotes pancreatic cancer cell growth

**DOI:** 10.18632/oncotarget.9009

**Published:** 2016-04-26

**Authors:** Noriyuki Omura, Masamichi Mizuma, Anne MacGregor, Seung-Mo Hong, Michael Ayars, Jose Alejandro Almario, Michael Borges, Mitsuro Kanda, Ang Li, Audrey Vincent, Anirban Maitra, Michael Goggins

**Affiliations:** ^1^ Department of Pathology, The Sol Goldman Pancreatic Cancer Research Center, Johns Hopkins Medical Institutions, Johns Hopkins University, Baltimore, MD, USA; ^2^ Department of Oncology, The Sol Goldman Pancreatic Cancer Research Center, Johns Hopkins Medical Institutions, Johns Hopkins University, Baltimore, MD, USA; ^3^ Department of Medicine, The Sol Goldman Pancreatic Cancer Research Center, Johns Hopkins Medical Institutions, Johns Hopkins University, Baltimore, MD, USA

**Keywords:** pancreatic cancer, ANK1, hypomethylation, ankyrin, mir-486

## Abstract

The methylation status of a promoter influences gene expression and aberrant methylation during tumor development has important functional consequences for pancreatic and other cancers. Using methylated CpG island amplification and promoter microarrays, we identified *ANK1* as hypomethylated in pancreatic cancers. Expression analysis determined *ANK1* as commonly overexpressed in pancreatic cancers relative to normal pancreas. *ANK1* was co-expressed with miR-486 in pancreatic cancer cells. Stable knockdown of *ANK1* in the pancreatic cancer cell line AsPC1 led to changes in cell morphology, and decreases in colony formation. Stable knockdown of *ANK1* also marked reduced the growth of tumors in athymic nude mice. Among patients undergoing pancreaticoduodenectomy, those with pancreatic cancers expressing *ANK1* had a poorer prognosis than those without *ANK1* expression. These findings indicate a role for *ANK1* overexpression in mediating pancreatic cancer tumorigenicity.

## INTRODUCTION

Pancreatic ductal adenocarcinoma is the third leading cause of cancer-related deaths in the USA, with an overall 1-year survival rate of <15% [1]. Pancreatic adenocarcinomas are locally invasive and highly metastatic and most patients have advanced stage disease at the time of diagnosis [2]. A better understanding of the molecular changes that mediate the invasive and metastatic properties of pancreatic cancer is needed in order to develop more effective therapies for the treatment of this deadly disease.

Aberrant CpG island methylation is an important cause of altered gene function in human cancer [3]. This altered gene function arises from both gene silencing associated with aberrant hypermethylation of promoter CpG islands and induction of gene expression associated with gene hypomethylation. DNA methylation abnormalities are thought to contribute to pancreatic cancer development and progression [4–13]. Several strategies have been used to identify differentially methylated genes in pancreatic cancers including analysis of expression changes after DNA methylation inhibition, array-based methods, and methylome sequencing [5, 6, 10, 11, 14, 15]. Using one such method that combines methylated CpG island amplification (MCA) and promoter microarray detection [14], we identified *ANK1* as a hypomethylated gene in pancreatic cancers.

*ANK1* is a member of the ankyrin family of membrane-associated cytoskeletal proteins expressed in a variety of biological systems [16]. Vertebrates have three ankyrins, *ANK1*, *ANK2*, and *ANK3*, each containing multiple alternatively spliced variants. Most ankyrin isoforms contain three functional domains: a conserved N-terminal ankyrin repeat domain (ARD); a spectrin binding domain; and a variably sized C-terminal regulatory domain. Many other proteins besides ankyrins have ankyrin repeat domains. *ANK1*, also known as erythroid ankyrin, is a large (~210 kD) multifunctional protein [17] that stabilizes the erythrocyte cytoskeleton and is defective in Hereditary Spherocytosis [18]. Small isoforms of *ANK1* have been found in skeletal and cardiac muscle and in brain [18]. Ankyrins mediate signaling events through the interaction of its ankyrin repeat domain with cell surface receptors such as CD44 [19, 20]. Recent studies have identified aberrant hypermethylation of *ANK1* in the brains of patients with Alzheimer's disease [21, 22].

We examined the expression and transcriptional regulation of *ANK1* in pancreatic adenocarcinomas and investigated its potential role in tumor progression.

## RESULTS

### ANK1 hypomethylation

**Figure 1:**
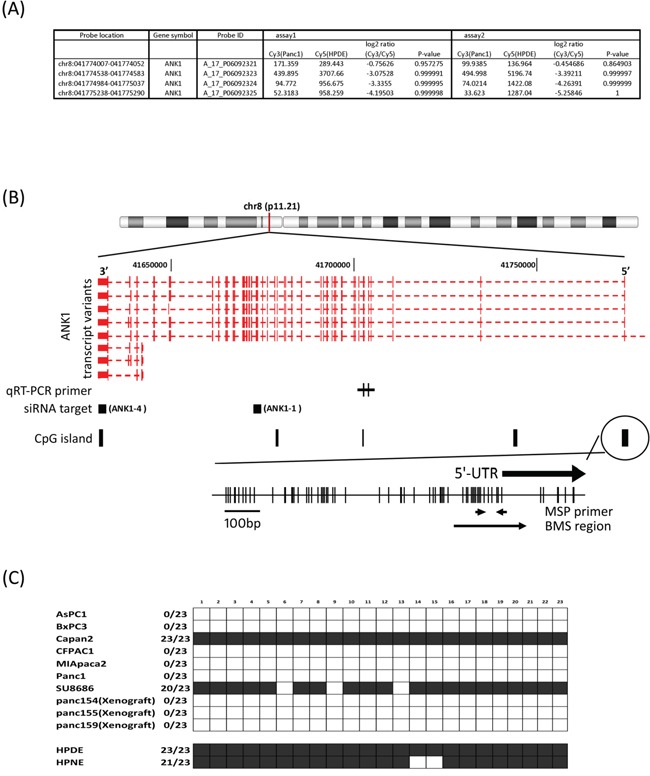
**A.** MCA array data. Cy5/Cy3 log_2_-fold change and p-value was obtained by Agilent's ChIP Analytics 1.3 software. **B.** Genetic map of the *ANK1* locus on chromosome 8q11.21 showing the location of transcripts, CpG islands, CpG sites, primer locations and siRNA target region. The quantitative RT-PCR primers are designed to recognize the large or small *ANK1* isoforms as indicated. The location of the shRNA target sequences are represented by black squares. There are 4 CpG-islands defined in the UCSC genome browser and primers for MSP and BMS are designed to determine the methylation status of the 5′flanking CpG island. **C.** Methylation status of 23 CpG sites in the ANK1 promoter region determined by bisulfite sequencing (BMS region in 1B). Black box: methylated; white box: unmethylated.

*ANK1* was identified as a candidate gene undergoing promoter methylation after comparing promoter methylation profiles of the pancreatic cancer line, Panc-1 and the non-neoplastic pancreatic epithelial cell line, HPDE by using MCA in conjunction with the Agilent 44K promoter array [14]. On the array three probes specific for the *ANK1* CpG-island had elevated log_2_ Cy3/Cy5 ratios (log_2_ >2) indicating hypomethylation in Panc-1 relative to HPDE (Figure 1A). We also examined DNA methylation patterns of *ANK2* and *ANK3* in our MCA array data and found no evidence for differential methylation (data not shown).

Since *ANK1* has not been previously shown to be aberrantly methylated in cancer, we further examined the methylation of *ANK1*.

We performed bisulfite modified sequencing (BMS) to verify hypomethylation of the *ANK1* promoter. The 5′-flanking region of the *ANK1* transcription start site has a large CpG island (899 bp; −861 to +39; %GC=58.6; observed CpGs/expected CpGs=0.722) and contains the consensus binding sites for GATA-1 and CACCC-binding proteins. Both transcriptional factors are thought to be essential for high-level expression of *ANK1* in erythrocytes [18]. Primers were designed to assess the methylation status of CpGs near the transcriptional start site (Figure 1B): 23 CpG sites were sequenced. Of 7 pancreatic cancer cell lines and 3 xenografts examined, 8 samples were completely unmethylated at these CpG sites, while non-neoplastic pancreatic cell lines and two pancreatic cancer cell lines (Capan2 and SU8686) were fully methylated (Figure 1c).

We next performed bisulfite sequencing of normal pancreas. For this experiment we amplified a 361 bp amplicon with BMS primers that included the CpGs amplified by MSP. We found partial methylation in 3 of 9 normal pancreata by BMS. The CpGs methylated in normal pancreata were located −159 to −132 nucleotides upstream of the ATG start site and included the region amplified by the MSP primers).

**Figure 2:**
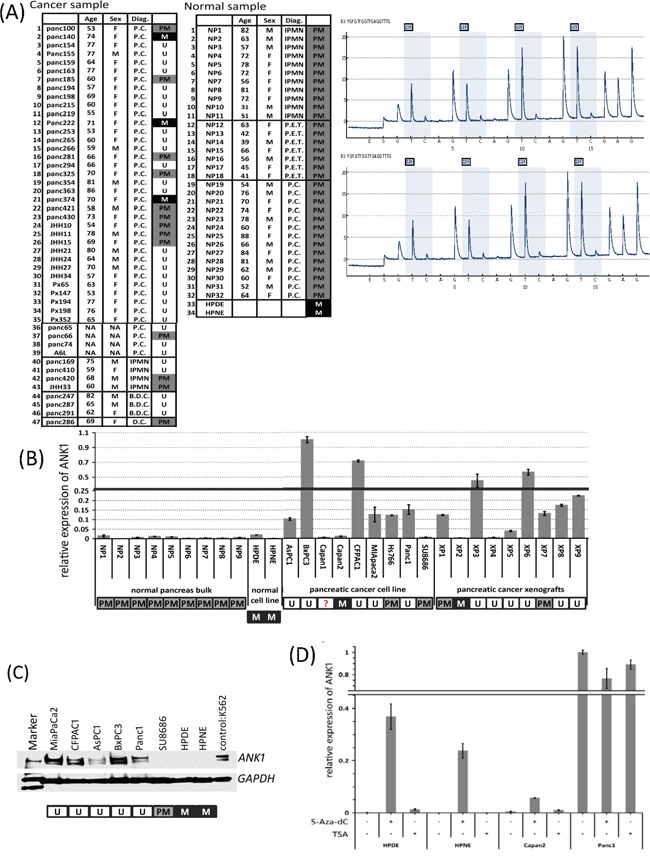
**A.** Methylation-specific PCR analysis of *ANK1* in normal and neoplastic cells or tissues. Key: M in black box: methylated; PM in grey box: partial methylation; U in white box: unmethylated; M=male; F=female. **B.** Expression of *ANK1* in normal and neoplastic tissues or cells. The bar graph summarizes qRT-PCR data analyzed by the delta-delta Ct method. Each Ct value was normalized to *GAPDH* and calibrated by the average of BxPC3 delta Ct value (=1). Data are the mean ± SD. **C.** Western blot of Ank1. The Ank1 protein appears as two major bands at approximately 210 kDa. The lysate from K562 cells was used as a positive control and *GAPDH* (~40kDa) was used as a loading control. **D.** Induction of *ANK1* mRNA by 5-aza-dC. In *ANK1* negative lines HPDE, HPNE and Capan2, *ANK1* expression was induced after 5-aza-dC treatment, but TSA had no effect. Each Ct value was normalized to *GAPDH* and calibrated by a reference to untreated Panc-1 cells. Data are shown as the mean ± SD.

We next employed pyrosequencing to further analyze the DNA methylation status of the ANK1 promoter in 5 normal pancreatic duct samples. This analysis confirmed that the ANK1 promoter was partially methylated, but predominantly unmethylated (10-15% CpGs analyzed were methylated) (Figure 2).

We next examined the methylation status of pancreatic tissue samples by methylation specific PCR (MSP). The MSP assay correlated exactly with results obtained by BMS in the 9 cell lines examined. We then expanded the panel for MSP analysis to 10 pancreatic cancer cell lines, 47 xenografts of primary pancreatic cancers, 2 normal pancreatic cell lines, 32 normal pancreatic tissues and 2 microdissected normal duct epithelia. As shown in Figure 2A, MSP analysis revealed that 30 of 47 (63.8%) cancer xenografts and 5 of 10 (50.0%) cancer lines were unmethylated while the normal samples were all partially methylated.

### ANK1 expression

Next we analyzed *ANK1* mRNA levels in a panel of pancreatic cancers and normal samples using quantitative RT-PCR. *ANK1* expression was detected in 6 of 9 pancreatic cancer cell lines and 7 of 9 xenografts but little or no expression was detected in normal pancreatic and liver tissues or in the non-neoplastic pancreatic cell lines, HPDE and HPNE (Figure 2B). We also examined *ANK1* expression in our Serial Analysis of Gene Expression (SAGE) database [23] and found *ANK1* was expressed in 18 (75%) of 24 pancreatic cancer samples but was not expressed in microdissected normal pancreatic duct or in the non-neoplastic cell line, HPDE. SAGE analysis also revealed that *ANK2*, the ankyrin expressed in lymphocytes, was not differentially expressed in pancreatic cancers.

Small isoforms of *ANK1* that are expressed from a different promoter have been described. These isoforms are more widely expressed and have a different structure and intracellular localization compared to the large isoform of *ANK1* [24]. However, our MCA data did not reveal any difference in methylation between pancreatic cancer and normal pancreas for the CpG island associated upstream of this small isoform (data not shown). We also examined the expression of small isoforms of *ANK1* using specific qRT-PCR primers (Figure 1B) and found no relationship between the expression of the small and large isoforms of *ANK1*.

We next examined the expression of ankyrin-1 protein by immunoblotting analysis. As shown in Figure 2C, the ankyrin-1 antibody detected two major bands around the expected size of 200 kDa in the control erythroleukemia cell line, K562 (Figure 1B) and in pancreatic cancer lines but not in non-neoplastic pancreatic cell lines. These results are consistent with the qRT-PCR results described above. Since our pancreatic cancer cell lines aberrantly expressed the large isoform of ankyrin-1, for the remainder of the text we use ankyrin-*1* to refer to this larger isoform.

Since *ANK1* expression of in pancreatic cell lines was correlated with aberrant hypomethylation of the *ANK1* promoter, we investigated DNA methylation and histone modification in the regulation of *ANK1* transcription. After 5-aza-dC treatment *ANK1* mRNA was induced in the *ANK1*-non-expressing cell lines (Figure 2D), but there was no significant change in the *ANK1*-expressing cell line, Panc-1 after 5-aza-dC or TSA treatment supporting the role of DNA methylation in silencing the *ANK1* promoter.

**Figure 3:**
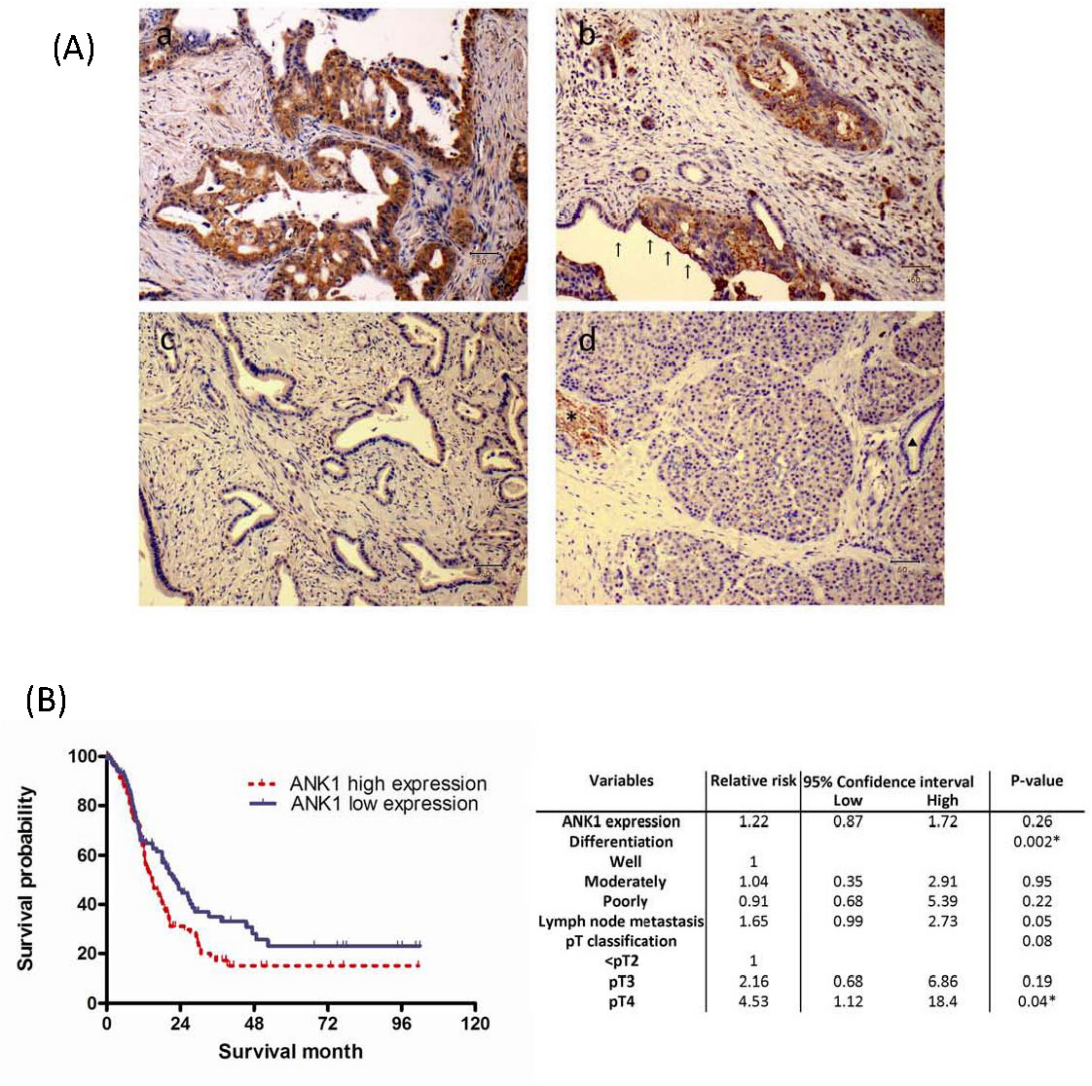
**A.** Ank1 immunohistochemical labeling in pancreatic ductal adenocarcinomas and normal pancreas. (a) Diffuse cytoplasmic and membranous Ank1 expression. Scattered strongly positive signal is observed in erythrocytes, which serve as an internal positive control. (b) Strong Ank1 labeling is identified in luminal border of as well as cytoplasm of tumor cells, while adjacent normal pancreatic ductal epithelial cells show lack of Ank1expression (arrows). (c) An example of a pancreatic cancer without Ank1 expression. (d) Ank1 was expressed in erythrocytes (*) with moderate intensity. Acinar cells and inflammatory cells are also diffusely and weakly stained to Ank1. In contrast, normal ductal epithelial cells are negative (triangle). **B.** Kaplan-Meier survival analysis of pancreatic ductal adenocarcinoma according to Ank1expression. Patients with high Ank1expression (median survival 28.0 months; n=125) had a significantly worse patients' survival time than those with low/no Ank1 expression (median survival 38.5 months; n=116; log-rank test, P=0.026).

Next, we performed immunohistochemical labeling of ankyrin-1 on TMAs containing primary pancreatic adenocarcinomas and adjacent non-neoplastic pancreatic tissues. Strong positive labeling was clearly detectable in erythrocyte membranes as expected (Figure 3A). Weak intensity labeling was also diffusely present throughout the cytoplasm in various cell types, including pancreatic acinar and stromal cells. Of 241 primary pancreatic cancers evaluated, 125 cases (51.9%) showed strong membrane immunolabeling for ankyrin-1 (Figure 3A) and/or diffuse labeling in the cytoplasm, whereas in the remaining 116 cases (48.1%) staining was either weakly present in the cytoplasm or completely absent (Figure 3A).

We found evidence that overexpression of ankyrin-1 had prognostic significance. There was a significant association between overexpression of ankyrin-1 and reduced survival, using univariate analysis (p=0.026, Figure 3B), with a median survival of 14.7 months in the ankyrin-1-overexpressing cases versus 22.6 months in patients without ankyrin-1- overexpressing cancers. But ankyrin-1 expression was also significantly associated with poorly differentiated cancers (p<0.04) and a multivariate analysis of prognostic factors associated with outcome of pancreatic cancer (including ankyrin-1 expression, tumor grade, lymph node metastasis, perineural invasion, vascular invasion and maximum tumor diameter) using the Cox proportional hazards model did not find that ankyrin-1 overexpression was an independent predictor of outcome (Relative risk, 1.22; P =0.26; Figure 3B).

**Figure 4:**
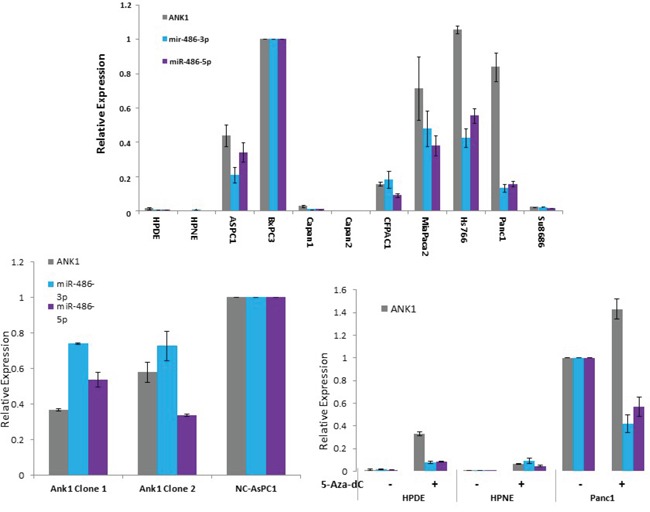
Co-expression of ANK1 and miR-486 in pancreatic cell lines **A.** Pancreatic cell line expression of ANK1, miR-486-3p and miR-486-5p. **B.** The effect of ANK1 knockdown on miR-486-3p and miR-486-5p expression. **C.** The effect of DNA methylation inhibition with 5-deoxycytosine on the expression of ANK1, miR-486-3p and miR-486-5p.

We also found that miR-486 is located in the coding region of *ANK1* suggesting that miR-486 is co-expressed with *ANK1* in pancreatic cancer cells. Consistent with this, miR-486 expression in erythrocytes is regulated by GATA1 binding to the *ANK1* promoter [25]. MiR-486 has been reported to be overexpressed in pancreatic cancer cells and in pancreatic precursor neoplasms [26, 27]. We examined the expression of miR-486-3p and miR-486-5p in pancreatic cancer cell lines and its relationship to ANK-1 expression and found a tight correlation, consistent with co-expression (Figure 4). To further examine this relationship, we examined miR-486 expression in cells with ANK1 knockdown and show that like ANK1, miR-486 expression is significantly reduced in these cells. We also examined the effect of DNA methylation inhibition on miR-486 expression on cells with methylation of the ANK1 promoter region that do not normally express ANK1 (HPDE and HPNE) as well as cells that normally express ANK1 (Figure 4B). We find that in HPDE and HPNE cells where both ANK1 and miR-486 are silenced, DNA methylation inhibition also induces expression of miR-486, whereas in Panc-1 cells which have an unmethylated ANK1 promoter region and expression of both ANK1 and miR-486 there was a mild reduction in miR-486 expression (Figure 4C).

### *ANK1* knockdown and anchorage-independent growth

**Figure 5:**
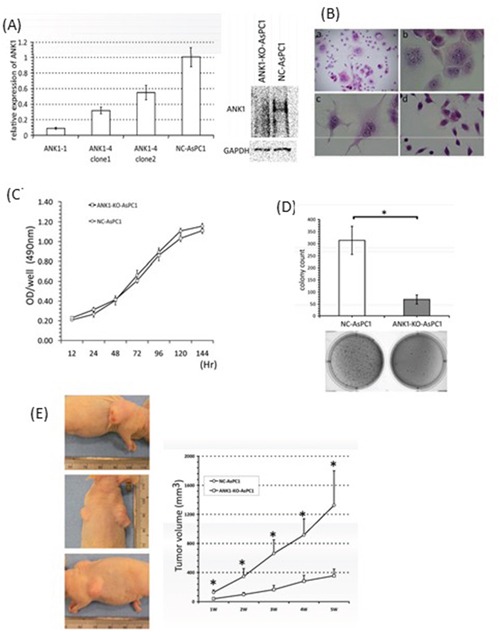
**A.** Analysis of *ANK1* expression in the knockout constructs. Expression was measured by qRT-PCR (left panel) and Western blotting (right panel). **B.** Morphology of pancreatic cancer cells with the *ANK1* knockdown. (a) and (b) a significant portion ANK1-4 cells had multiple nuclei and the associated cytoplasm was markedly enlarged. (c) The same phenotype was present in the other knockout line, ANK1-1. (d) Negative control line NC-AsPC1: Original magnification; (a) ×40 (b)-(d) ×200; H & E staining. **C.** Anchorage-dependent cell growth quantified by MTS assay. No significant change in the growth rate can be seen between ANK1-KO-AsPC1 and NC-AsPC1 cells. **D.** Anchorage-independent cell growth was measured by soft-agar colony formation assay. A bar chart shows the mean value (±SD) for colony counts in a defined field. Knockdown of *ANK1* significantly inhibits colony formation (*: p <0.001). **E.** Tumor formation in nude mice. ANK1-KO-AsPC1cells are injected in left flank of mouse and NC-AsPC1 cells are in right flank. Tumors in a representative animal containing NC-AsPC1 cells and ANK1-KO-AsPC1cells are shown in the left panel. Upper panel showed NC-AsPC1 tumor and lower panel showed ANK1-KO-AsPC1 tumor.

Next, we determined the consequences of reducing *ANK1* expression by stably expressing *ANK1*-targeted shRNAs. Two independent shRNA species, ANK1-1 and ANK1-4, efficiently reduced *ANK1* expression by 60-80% in AsPC1 cells 48 hours after transfection, while a non-silencing control shRNA had no significant effect (NC-AsPC1, Figure 5A). Both the ANK1-1 and ANK1-4 transfectants were grown under selection and pooled populations of clones were used for further studies (ANK1-KO-AsPC1). We observed ~70% reduction in *Ankyrin-1* protein in ANK1-KO-AsPC1 cells by immunoblotting (Figure 5A, right panel).

Clones isolated from ANK1-KO-AsPC1 cultures were stained with H&E (Figure 5B). Interestingly, changes in cell shape were observed after transfection with shRNAs, and some cells exhibited a markedly altered morphology, characterized by giant cells with multiple nuclei. Additionally, most of the nuclei in ANK1-KO-AsPC1 cells were slightly larger and had more frequent chromosome condensations than the NC-AsPC1 control cells (Figure 5B b-d). The same morphological change was produced in both independent stable knockdown lines transfected with ANK1-4 or ANK1-1 (Figure 5B b and c), suggesting that these changes are not due to any off-target effect of the siRNA.

The impact of reduced *ANK1* expression on cell proliferation was measured in the *ANK1* knockdown and control cells, but no difference was observed (Figure 5C). However, *ANK1* knockdown significantly decreased colony formation in soft agar compared to controls (Figure 5D, p<0.0001). Also, the tumorigenicity of ANK1-KO-AsPC1 cells was remarkably suppressed in athymic nude mice compared to control cells (Figure 5E, p<0.005) with reduced tumor volumes apparent within one week of transplantation (p=0.00029).

## DISCUSSION

The present study demonstrates that *ANK1* is aberrantly expressed in pancreatic adenocarcinomas in association with promoter hypomethylation and silencing its expression in pancreatic cancer cells is associated with phenotypic changes including decreases in *in vivo* tumor growth.

We found partial methylation of the *ANK1* promoter and no expression by immunohistochemistry in normal pancreatic ductal epithelia. Our results indicate that normal pancreas shows partial methylation of *ANK1* with pyrosequencing results indicating low-level promoter methylation (10-15% of CpGs at a locus). Since 5-aza-dC treatment of *ANK1*-silenced pancreatic cancer cell lines induces *ANK1* expression, promoter methylation is an important regulator of *ANK1* expression. We also found evidence that miR-486 which is located in the *ANK1* coding region was co-expressed with *ANK1*. Prior studies have found that miR-486 promotes growth of myeloid cells [25, 28], raising the possibility that its co-expression with *ANK1* could also promote pancreatic cancer growth. Although miR-486 is overexpressed in pancreatic cancer [26, 27], it likely has a limited role as a circulating diagnostic marker because of the abundant expression of miR-486 in erythrocytes.

We found that stable knockdown of *ANK1* expression induced [1] morphological changes in some cells, characterized by large cells with multiple nuclei, and [2] a significant decrease in growth in soft agar *in vitro*, as well as tumorigenicity *in vivo*. The presence of large, multinucleated cells in the *ANK1* knockdowns suggests that loss of *ANK1* can disrupt the machinery responsible for cell division, possibly through an effect on actin-filament rearrangement. Previous studies have suggested that ankyrin is a component of the spectrin-actin cytoskeleton and is involved in cell motility, activation, proliferation and the maintenance of specialized membrane domains [19]. Overall, our results indicate the potential value of downregulating *ANK1* as a strategy for inhibiting the tumorigenicity of pancreatic cancer cells.

In summary, we find that *ANK1* is overexpressed in the majority of pancreatic adenocarcinomas in association with DNA hypomethylation and promotes growth in soft agar and tumor growth *in vivo*. Ankyrin-1 may be an attractive therapeutic target for pancreatic cancers.

## MATERIALS AND METHODS

### Cell lines and tissue samples

Ten human pancreatic cancer cell lines AsPC1, BxPC3, Capan2, CFPAC1, MiaPaCa2, Hs766T, Panc-1, SU8686, Panc2.5, and Panc1.28 were maintained under standard conditions as was the and the immortalized pancreatic duct cell line, HPDE and the human pancreatic Nestin-expressing cells (HPNE) were generously provided by Dr Ming-Sound Tsao (University of Toronto) and Dr. Ouellette (University of Nebraska Medical Center, NE), respectively.

Normal and neoplastic tissues were obtained from pancreatic adenocarcinomas resected at the Johns Hopkins Hospital. Normal pancreata were obtained from discarded stored frozen tissues from 11 patients who underwent a pancreatic resection for an intraductal papillary mucinous neoplasm (IPMN); from 7 patients with a neuroendocrine tumor; and from 14 patients with invasive ductal adenocarcinoma. These 32 patients were of similar age to the patients with pancreatic adenocarcinoma (mean age 63.6 years). The demographic breakdown was 18 females, 31 Caucasians and 1 African-American. The normal pancreas from these patients was chosen to test for possible age-related or field-effect changes in methylation. Normal pancreatic tissue from each case was dissected for DNA isolation. Genomic DNA was isolated from 47 cancer xenografts, established from primary carcinomas as described (44). These xenografts consisted of 43 pancreatic cancers, 3 distal common bile duct cancers, and 1 duodenal cancer. The mean age of this group was 67.0 years (30 females, 37 Caucasians, 4 African-Americans, 2 Asians, and 4 of unknown ethnicity). Formalin-fixed paraffin-embedded tissues were retrieved from 252 patients who underwent surgical resection at our institution to create tissue microarrays (TMAs) for immunohistochemical analyses. All specimens were collected and analyzed with the approval of the Johns Hopkins Committee for Clinical Investigation.

### Methylated CpG island amplification and (MCA) microarray analysis

Microarray data for Panc-1 versus HPDE cells was examined using data we previously generated [14], using Agilent's Human promoter 44K chip-on-chip microarray (Agilent Technologies). Methylation-specific sites were called with a statistical p-value and log2 ratio using Agilent's ChIP Analytics 1.3 software, incorporating the Whitehead Error Model.

### Bisulfite-modified sequencing and methylation-specific PCR

The methylation status of the *ANK1* 5′ CpG-islands was determined by bisulfite-modified sequencing (BMS) and methylation-specific PCR (MSP) as described previously [14]. Primers for *ANK1* BMS and MSP are listed in Supplemental Table 1. Two sets of BMS primers were used with the shorter primers (primer set 2) used for pancreas tissue analysis. Sanger sequencing was performed at the Johns Hopkins Sequencing Facility as previously described [14]. Pyrosequencing was performed using primers targeting the ANK1 promoter as previously described [29].

### Treatment with 5-aza-2′-deoxycytidine (5Aza-dC) and trichostatin a (TSA)

Cells were treated with 5-aza-dC (Sigma Chemical) at 1μmol/L for 4 days and/or 1μmol/L of TSA for 24 hours as previously described [5]. Total RNA from frozen tissues or cell lines was extracted using the mirVana miRNA Isolation Kit (Ambion) and processed using the DNA-free Kit (Ambion) to eliminate DNA.

### Real-time reverse transcription polymerase chain reaction (real-time RT-PCR)

2μg of total RNA was reverse transcribed using Superscript III and 250 ng of random hexamers (Invitrogen Life Technologies). The cDNA of *ANK1* was quantified using the SYBR green I method as previously described [30].

Primers for *ANK1* qRT-PCR are shown in supplemental Table 1. The housekeeping genes *GAPDH* and *PGK1* were used as references. Real time RT-PCRs were performed in triplicate as previously described [30].

### Immunohistochemistry

The HRP EnVision^+^ System (DAKO Corp.,) was used to evaluate ankyrin-1 protein expression in TMAs using a mouse polyclonal anti-ankyrin antibody (ABR-Affinity Bioreagents; 1:1000 dilution) using methods previously described (30). Erythrocytes were used as a positive control. The relative intensity of labeling was evaluated in neoplastic and normal duct cells. The area of immunostaining was scored as follows: 0, 0 to 5% of labeled tumor cells; 1, 5% to 25%; 2, from 25% to 50%; and 3, from 50 to 75%; 4, above 75%. Staining intensity was scored as follows: 0, no appreciable labeling; 1, mild; 2, strong intensity. The scoring index was determined by multiplying the area score and the intensity score.

### Generation of a stable ANK1 knockdown cell line

The antisense constructs were purchased from Open Biosystems (Huntsville, AL). They included *ANK1*-shRNA constructs ANK1-4 (Cat# V2HS_71234) and ANK1-1(Cat#V2HS_89652); the non-silencing negative control shRNA (Cat# RHS1707); and the empty vector control pSM2 (Cat# RHS1704). To establish a stable *ANK1*-knock down cell line, each vector was transfected into AsPC1 cells with Effectene (Qiagen) and 1~2μg/ml puromycin added to the media 48 hr after transfection. Selection was carried out for 2~3 weeks and puromycin-resistant cells were cloned by limiting dilution, in 96-well tissue culture plates. Clones were expanded in 24-well plates then checked for *ANK1* expression by qRT-PCR and western blotting. Pooled populations of 5~10 clones were used to make the *ANK1* knockdown line ANK1-KO-AsPC1 and the negative control line (NC-AsPC1).

### Western blotting

Total protein lysates were extracted in RIPA buffer (Roche Diagnostics, Indianapolis, IN). Protein concentrations were determined using the Bio-Rad protein assay system (Bio-Rad Laboratories Hercules, CA). Membranes were then incubated overnight at 4°C with mouse polyclonal anti-ankyrin-1 (ABR-Affinity Bioreagents) or rabbit polyclonal anti-*GAPDH* (Sigma). Membranes were incubated with horseradish peroxidase (HRP)-conjugated secondary antibody in 2.5 K% dry milk for 1 hr. The anti-mouse antibody was diluted 1:1000 and the anti-rabbit antibody was diluted 1:2000 (Amersham Inc, NJ). Bound antibody was detected with the ECL system (GE Healthcare, Piscataway, NJ).

#### Cell proliferation and colony formation assays

For cell proliferation assays, 1×10^4^ cells were plated into 96-well plates and proliferation measured with the MTS assay (Promega)) according to the manufacturer's instructions.

To measure anchorage-independent growth, 1×10^5^ cells /well were suspended in 0.35% agar and layered into 6-well plates containing 0.8% agar. Cells were allowed to grow for 2 weeks and spherical colonies were photographed using Chemi Doc XRS (Bio-Rad) and counted with Quantity One 1-D Analysis Software (Bio-Rad).

#### Mouse xenograft studies

Six-week-old male nu/nu mice were injected with 5 × 10^6^ ANK1-KO-AsPC1 and NC-AsPC1 cells in a volume of 200 μL of PBS/Matrigel (1/1 (v/v). Injections were made subcutaneously in the left and right flanks, respectively at day 0. Tumor volume was measured using electronic calipers weekly as previously described [31]. All animal experiments conformed to the guidelines of the Animal Care and Use Committee of Johns Hopkins University. Animals were maintained in accordance with the guidelines of the American Association of Laboratory Animal Care.

### Statistical analysis

Values reported are means ± SD. All data were normally distributed and underwent equal variance testing. Statistical analysis was performed using the SPSS program, (v. 11.0.1J). Mean differences between two subgroups were compared using Student's *t*-test. P<0.05 was considered statistically significant.

## SUPPLEMENTARY TABLE


