# Supplementary material for: Overexpression of ankyrin1 promotes pancreatic cancer cell growth
Source: Oncotarget. 2016 Apr 26;7(23):34977–87. doi: 10.18632/oncotarget.9009 (PMC5085203; doi:10.18632/oncotarget.9009)
Supplement: Supplementary file 1 [file oncotarget-07-34977-s001.pdf]

Supplemental Table 1

**Bisulfite modified sequencing**		
ANK1	Forward	5’-GAGGAGGTGTTTTTGTAATTTG-3’
	Reverse	5’-CACCTACTACCTTCAACTAC-3’
	Sequencing	5’-ACCTAAATAACCCCCTCCTAACATCTC-3’
**Methylation specific PCR**		
ANK1	M-MSP (F)	5’-GAGCGTTCGGTTCGATAG-3’
	M-MSP (R)	5’-TACGAAACCTATAACGTACG-3’
	U-MSP (F)	5’-GGGAGTGTTTGGTTTGATAGT-3’
	U-MSP (R)	5’-CTCTACAAAACCTATAACATACA-3’
**Quantitative RT-PCR**		
Large ANK1	Forward	5’-GGCCCGCAACGACGACACG-3’
	Reverse	5’-AATGTGCAGGGGCGTGAATC-3’
Small ANK1	Forward	5’-TCAGGGGGTCCCTGTGCTTTGT-3’
	Reverse	5’-GCCCTGCTCATCCGTGAATTGC-3’
GAPDH	Forward	5’-CCATGGAGAAGGCTGGGG-3’
	Reverse	5’-CAAAGTTGTCATGGATGACC-3’
PGK1	Forward	5’-CTTCCAGGAGCTCCAAACTG-3’
	Reverse	5’-GCTCTCATGGATGAGGTGGT-3’
